# Establishment and Advances of Third-Generation Hybrid Rice Technology: A Review

**DOI:** 10.1186/s12284-023-00670-z

**Published:** 2023-12-08

**Authors:** Zhufeng Chen, Jianxin Wu, Xing Wang Deng, Xiaoyan Tang

**Affiliations:** 1 Center for Biological Science and Technology, Key Laboratory of Cell Proliferation and Regulation Biology of Ministry of Education, Zhuhai-Macao Biotechnology Joint Laboratory, Advanced Institute of Natural Science, Beijing Normal University, Zhuhai, 519087 China; 2 Guangdong Provincial Key Laboratory of Biotechnology for Plant Development, School of Life Sciences, South China Normal University, Guangzhou, Guangdong 510631 China; 3 Shenzhen Institute of Molecular Crop Design, Shenzhen, 518107 China

**Keywords:** Rice, Hybrid breeding, Genic male sterility, Third-generation hybrid rice technology

## Abstract

Rice (*Oryza sativa* L.) is one of the most important food crops worldwide. The utilisation of heterosis (hybrid vigour) has played a significant role in increasing rice yield and ensuring food supply. Over the past 50 years, the first-generation three-line system based on cytoplasmic male sterility, and the second-generation two-line system based on environment-sensitive genic male sterility (EGMS), have been widely applied in hybrid rice production. However, the three-line system is restricted by the matching relationship among the three parental lines and allows only ~ 2–5% of germplasms to be explored for elite combinations. The environmental sensitivity of EGMS lines has posed serious risks to the production of hybrid seeds. These factors have hindered the development and applications of hybrid rice. Third-generation hybrid rice technology (TGHRT) is based on environment-insensitive genic male sterility, which can effectively overcome the intrinsic problems of the three-line and two-line systems. Since the establishment of TGHRT, numerous findings and innovations have been reported. This paper gives a brief review of traditional hybrid rice technologies and discusses the establishment of TGHRT, technical innovations in TGHRT, and future research that is necessary to promote the wide application of TGHRT in rice production.

## Introduction

Heterosis, also known as hybrid vigour, is the phenomenon of two genetically distinct parents producing an F_1_ hybrid that surpasses the parental lines in multiple phenotypic characteristics (Birchler 2015). Harnessing heterosis is an important strategy to improve the yield and broaden the adaptability of many crops. The phenomenon has been widely exploited in the breeding of many crops, such as maize (*Zea mays*), rice (*Oryza sativa*), cotton (*Gossypium herbaceum*), sorghum (*Sorghum bicolor*), foxtail millet (*Setaria italica*), rapeseed (*Brasicca napus*), sesame (*Sesamum indicum*), tomato (*Solanum lycopersicum*), bell pepper (*Capsicum annuum*), cucumber (*Cucumis sativus*), courgette (*Cucurbita pepo*), watermelon (*Citrullus lanatus*), and melon (*Cucumis melo*) (Bohra et al. 2016).

Efficient production of hybrid seeds at a commercial scale is crucial for the application of heterosis in agriculture. Maize is an important crop with separate male and female flowers on the same plant. Hybrid seeds of maize can be produced by pollinating the ear of the female parent with pollen from the male parent after the tassel of the female parent is manually or mechanically removed. As early as the 1920s, maize became the first hybrid crop to be commercialised because hybrid seed production from maize was relatively simple and convenient (Duvick 2001). Most diclinous gourd crops (such as pumpkins, loofah, and cucumber) and monoclinous crops with large floral organs (such as cotton, tomato, and pepper) are manually emasculated to facilitate cross-pollination. However, manual or mechanical emasculation is impractical for the large-scale production of hybrid seeds for monoclinous crops that have relatively small reproductive organs, such as rice, wheat, and rapeseed (Longin et al. 2012). Therefore, the development of male-sterile lines is crucial for these crops.

Rice is an important cereal crop worldwide. Strong hybrid vigour has been demonstrated in rice, leading to a yield increase of approximately 20% in the F_1_ hybrid compared with the parental lines (Cheng et al. 2007; Ma and Yuan 2015). Hybrid rice has been commercialised in China since the 1970s, and it has significantly contributed to rice production and food supply domestically and globally (Li and Yuan 2010). Currently, two technologies are widely used in hybrid rice production: the three-line method and the two-line method. The former utilises cytoplasmic male sterility (CMS) and is known as first-generation hybrid rice production. The latter deploys environment-sensitive genic male sterility (EGMS) and is known as second-generation hybrid rice production (Wang and Deng 2018).

**Fig. 1:**
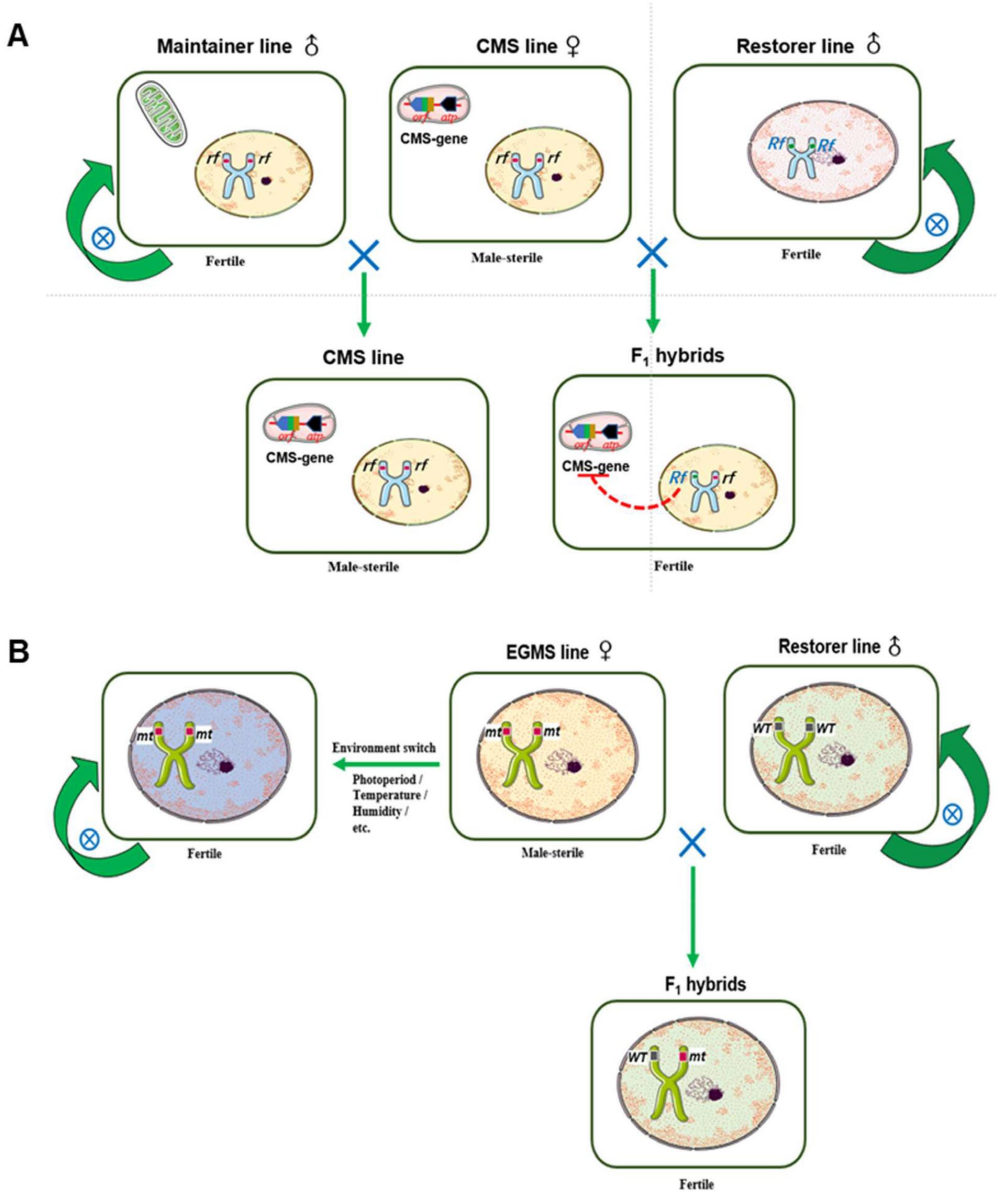
Three-line system (the first-generation hybrid rice) and two-line system (the second-generation hybrid rice) for rice breeding. **(A)** The three-line system. The cytoplasmic male sterility (CMS) line, which carries the mitochondrial CMS gene, but does not have a functional nuclear restorer gene (*Rf*), is propagated by cross-pollination by the maintainer line. The *Rf* genes encode proteins that are targeted to mitochondria to inhibit the CMS gene, thus restoring the fertility of the F_1_ hybrid. **(B)** The two-line system. The environment-sensitive genic male sterility (EGMS) line carries a recessive nuclear EGMS gene (*mt*). The restorer line carries the wild-type EGMS gene (*WT*). Under restrictive conditions (such as long day length, high temperature, or low humidity), the EGMS line is male-sterile and can cross with the restorer line to produce F_1_ hybrid seeds. Under permissive conditions (such as short day length, low temperature, or high humidity), the EGMS line can produce functional pollen and self-pollinate. The restorer line can be any normal rice germplasm that can self-reproduce

The three-line method requires a CMS line, a maintainer line, and a restorer line (Chen and Liu 2014). The CMS line contains a cytotoxic mitochondrial gene that inhibits the production of fertile pollen. Many CMS genes have been identified. Typically, they are chimeric genes created by rearrangement, insertion, and/or deletion of the mitochondrial genome (Tang et al. 2017). A CMS line cannot produce offspring through self-pollination, but can produce progeny by accepting pollen from either the maintainer line or the restorer line (Fig. 1A). The maintainer line has the same nuclear genome as the CMS line, but without the CMS gene, and its pollen exhibits normal fertility. The offspring produced by the CMS line after cross-pollination by the maintainer line retains all the characteristics of the CMS line, thus achieving propagation of the CMS line (Fig. 1A). The restorer line is different from the CMS line in its nuclear genome, and it contains restorer (*Rf*) gene(s) that can inhibit the function of the CMS gene. When the restorer line is crossed with the CMS line, a fertile hybrid species with hybrid vigour is produced (Fig. 1A). Thus far, eight types of CMS have been identified in rice: Wild-Abortive (CMS-WA), Indonesian Shuitiangu (CMS-ID), K52(CMS-K), Gambiaca (CMS-G), Dissi (CMS-D), Dwarf Abortive (CMS-DA), Honglian (CMS-HL), and Boro II (CMS-BT) (Chen and Liu 2014). Among them, CMS-WA, which was originally identified in a plant of Asian common wild rice (*Oryza rufipogon*) in Hainan, China, has been widely used in hybrid rice breeding and accounts for the majority of the released three-line hybrid varieties. According to the Ministry of Agriculture and Rural Affairs of the People’s Republic of China, the planting area of three-line hybrid rice reached 4.9 million hectares in China in 2015, accounting for 51.6% of the total hybrid rice acreage in China (Bai et al. 2018).

Despite the huge success of the three-line system, the poor genetic diversity of the CMS lines and unreliable fertility restoration of the F_1_ hybrids have limited the breeding of superior hybrid varieties (Dai et al. 2016; Abbas et al. 2021). *Rf* genes exist in only 2–5% of rice germplasms (Wang and Deng 2018). Thus, only a small proportion of rice germplasms can be used in pairing tests, and the vast majority of germplasm resources cannot not be exploited for heterosis (Peng et al. 2009). In addition, it is challenging to breed new traits into a CMS line. To breed a new CMS line, it is essential to first breed its corresponding maintainer line. The maintainer line is then used as the recurrent parent to repeatedly backcross with an existing CMS line until the nuclear genome of the old CMS line is replaced by the maintainer genome (Ren et al. 2016). This process takes much longer and requires much more work than breeding a regular rice cultivar.

The two-line system consists of an EGMS line carrying a recessive EGMS gene and a paternal line with a different nuclear genome (Wang et al. 2001; Zhang et al. 2014). The EGMS line can function as either the maintainer line or the male-sterile line depending on the environmental conditions (Fig. 1B). Under restrictive conditions (e.g., long days, high temperature, or low humidity), EGMS lines are male-sterile and can cross with the paternal lines to produce hybrid seeds. Under permissive conditions (e.g., short days, low temperature, or high humidity), EGMS lines are male-fertile and can self-reproduce (Fig. 1B). At least 15 EGMS genes have been identified in rice (Peng et al. 2022). Several EGMS genes have been cloned, including the photoperiod-sensitive male-sterility genes *PMS1* (Fan et al. 2016) and *PMS3* (Ding et al. 2012b); the thermosensitive male-sterility genes *TMS5* (Zhou et al. 2014), *TMS10* (Yu et al. 2017) and P/*TMS12-1* (Zhou et al. 2012; Ding et al. 2012c); and the reverse photoperiod-sensitive male-sterility gene *CSA* (Zhang et al. 2013). *PMS1* and *PMS3* encode a phased small-interfering RNA and a long noncoding RNA, respectively, and both *pms1* and *pms3* mutants are responsive to both photoperiod and temperature (Ding et al. 2012a, b; Fan et al. 2016). *TMS5* encodes an *RNaseZ*^*S1*^ that directly and specifically cleaves ubiquitin-ribosomal L40 (*Ub*_*L40*_) family mRNAs (Zhou et al. 2014). The excessive accumulation of *Ub*_*L40*_ mRNAs induced by high temperature causes male sterility of the *tms5* mutant. Because the environment-sensitive male-sterile phenotype is controlled by a recessive gene, all regular rice germplasms can serve as the paternal line to produce fertile F_1_ hybrids. The two-line system has distinct advantages over the three-line system, including increased pairing flexibility, complete utilisation of heterotic resources, simplified breeding procedures and shortened breeding cycles for EGMS lines, and simplified propagation of male-sterile lines (Huang et al. 2014; Ashraf et al. 2020). By 2015, the annual cultivation area of two-line hybrid rice in China reached 4.59 million hectares, accounting for 48.4% of the total land area devoted to hybrid rice (Zhang et al. 2023b). At present, the two-line hybrid method is the main method for exploiting heterosis in rice (Yuan 1990; Ma and Yuan 2015; Pak et al. 2021).

Despite all of its advantages, the two-line method also has intrinsic problems. EGMS lines may become partially male-fertile and self-pollinate during abnormal fluctuations of ambient conditions, which causes impurity of the F_1_ hybrid seeds (Ashraf et al. 2020). In addition, because environment-sensitive sterility is regulated not only by the major EGMS gene, but also by several other minor genes, EGMS lines with different genetic backgrounds often display significant variations in critical sterility-inducing temperature (the threshold temperature at which an EGMS line changes from fertile to completely sterile). Furthermore, the critical temperature of the EGMS line often increases after several generations of propagation. The environment-sensitive nature of EGMS lines poses severe risks to the propagation of EGMS lines and the production of pure F_1_ hybrid seeds (Lei et al. 2013).

## Proposal and Establishment of Third-Generation Hybrid Rice Technology

Many male-sterile mutants in flowering plants are regulated by genes that are insensitive to environmental conditions, which is known as genic male sterility (GMS). GMS is usually caused by recessive mutation of nuclear genes. Numerous GMS materials and their corresponding functional genes have been identified. These genes are involved in various processes, such as meiosis during anther and pollen development, callose metabolism, the development and apoptosis of the tapetum, polysaccharide and lipid metabolism, transport of lipids and proteins, and formation of the pollen exine (Wan et al. 2020; Abbas et al. 2021; Tariq et al. 2022). Because GMS is controlled by a recessive nuclear gene, any rice germplasm containing the wild-type gene can restore the fertility of the F_1_ hybrid. The GMS system offers a wider selection of germplasms as paternal lines for breeding hybrids of strong heterosis. Additionally, the pollen sterility of GMS material is stable, and thus, it is safe for hybrid seed production. GMS material is especially valuable for addressing the problems associated with the first two generations of hybrid rice technology (Wang and Deng 2018). However, the application of GMS material to heterosis has been hindered by the difficulty of propagating pure GMS lines.

To address this problem, in 1993, researchers proposed creating a transgenic maintainer for a recessive GMS plant by transforming it with two linked genes, namely the male fertility-restoration gene and a pollen-inactivation gene. Pure GMS seeds can be propagated by pollinating the male-sterile lines with this maintainer (Williams and Leemans 1993). Later in 2002, Perez-Prat and van Lookeren Campagne (2002) presented two alternative strategies. One was to link the fertility-restoration gene with a seed-colour gene and transform it into the GMS mutant to obtain a colour-maintainer line. Cross-pollination of the colour-maintainer with the male-sterile line would generate 50% male-sterile seeds and 50% colour-maintainer seeds that could be separated based on the seed colour. The other was to link the fertility-restoration gene with a seed-colour gene and a pollen-inactivation gene and transform it into the GMS mutant to produce a maintainer line. Self-pollination of this maintainer would produce two types of seeds that could be differentiated based on colour: 50% colour-less male sterile seeds, and 50% coloured maintainer seeds. Cross-pollination of the GMS line by the maintainer line would produce the pure GMS line (Perez-Prat and van Lookeren Campagne 2002).

Inspired by these ideas, researchers at Dupont Pioneer developed seed production technology (SPT) by transforming the male-sterile mutant *ms45* with the wild-type gene *MS45* linked with the maize α-amylase gene *ZmAA1* and the red florescence protein gene *DsRed2* (Wu et al. 2016). The *ZmAA1* gene inactivates pollen by disrupting starch formation in the pollen grain, while the *DsRed2* red fluorescence protein gene directs the production of red fluorescence in the transgenic seed. The resulting transgenic plant with one copy of the transgene (e.g., the SPT maintainer line) can generate two types of pollen grains: 50% are non-transgenic and fertile and the other 50% carry the SPT transgene and are infertile. As the transgene used in SPT has no effect on the female organ, self-pollination of the transgenic plant produces a 1:1 ratio of seeds with and without the transgene. The transgenic seeds display red fluorescence, while the non-transgenic seeds do not. The two types of seeds can be mechanically separated based on their colour, with the non-transgenic seeds representing the male-sterile line and the transgenic seeds representing the maintainer line. The SPT system has been used in hybrid maize breeding and production in the United States since 2012. It is regarded as non-transgenic by regulatory authorities in the United States, Australia, and Japan (Wu et al. 2016; Wang and Deng 2018). Nonetheless, because manual or mechanical emasculation has already been well-established in hybrid maize production practices, SPT has not been widely adopted because it does not have clear commercial advantages.

**Fig. 2:**
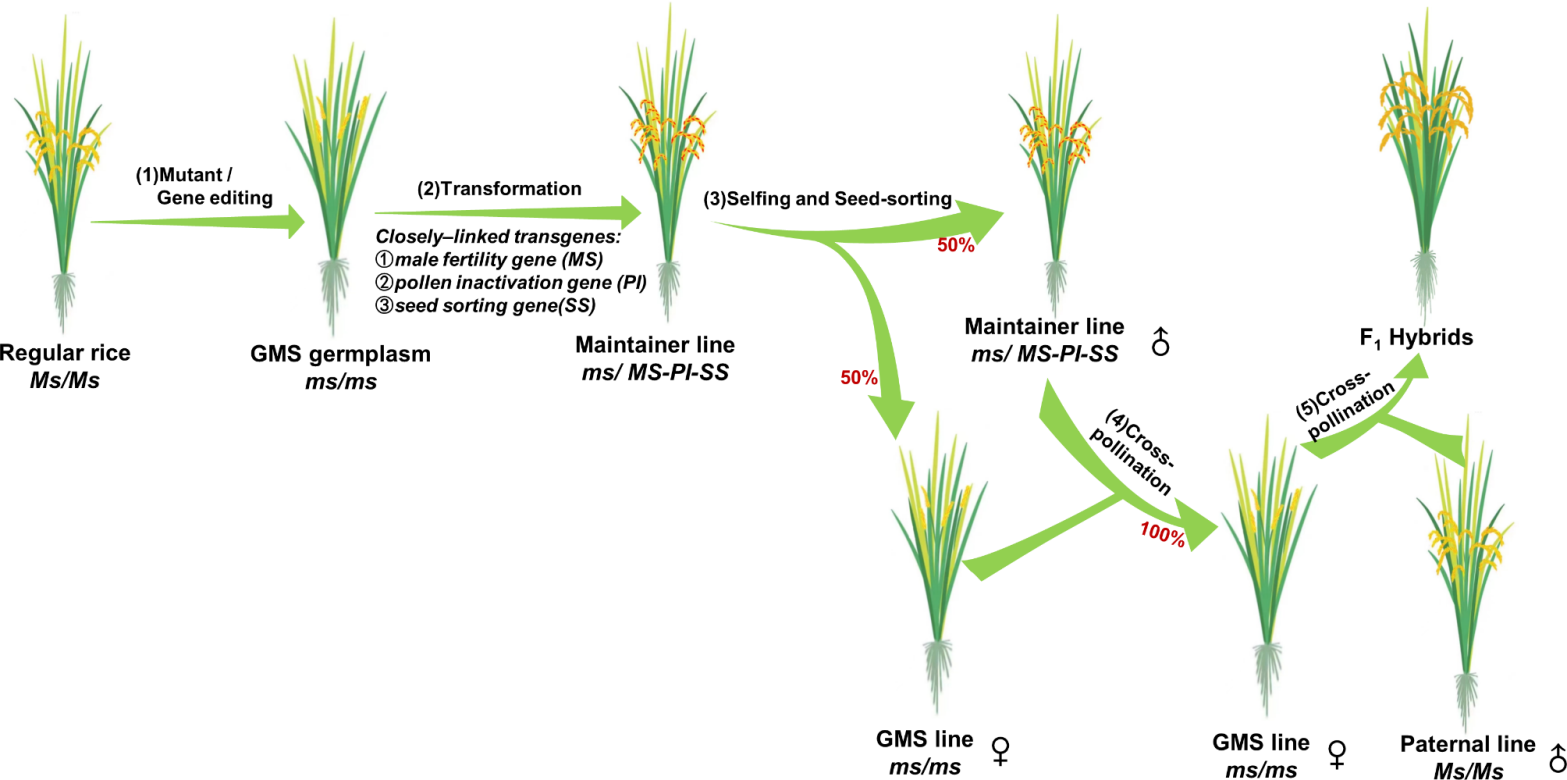
The third-generation hybrid rice technology. (1) Male-sterile lines are obtained by mutant screening or CRISPR/Cas9 technology. (2) The fertility-restoration gene, pollen-inactivation gene, and seed-sorting gene are connected in one T-DNA and transformed into the male-sterile plant to obtain a maintainer line. (3) The maintainer line self-pollinates and produces 50% male-sterile seeds and 50% maintainer seeds that are separated based on the seed-sorting marker. (4) Cross-pollination the male-sterile line by the maintainer generates 100% male-sterile seeds, because transgenic pollen is killed by the pollen-inactivation gene. (5) Any normal rice germplasm can be used as the paternal line to cross with a genic male sterility (GMS) line to breed hybrid rice

To develop an SPT system that can be applied to the commercial breeding of hybrid rice varieties, Chang et al. (2016) screened a mutant library of the *indica* variety Huanghuazhan (HHZ) and obtained the GMS mutant *osnp1.* This mutant exhibited normal vegetative growth, high stigma exsertion, a high out-crossing rate, and stable male sterility under various environmental conditions, all of which are desirable traits for hybrid seed production. Using the genome resequencing method (Yan et al. 2017), they cloned the novel gene *OsNP1*, which is specifically expressed in the tapetum and microspores (Chang et al. 2016). They then connected the wild-type *OsNP1* gene, the pollen-inactivation gene *ZmAA1*, and the red fluorescence seed-marker gene *DsRed2* in one T-DNA cassette and introduced it into the *osnp1* mutant, resulting in the creation of the maintainer line Zhen18B with a single copy of the transgene (Fig. 2). When self-pollinated, Zhen18B produced a 1:1 ratio of non-transgenic male-sterile seeds (Zhen18A) and transgenic fertile seeds (Zhen18B), which could be sorted mechanically using a fluorescence sorter. When Zhen18A was pollinated by Zhen18B, a large quantity of high-purity non-transgenic male-sterile seeds was produced (Fig. 2). To evaluate the potential of Zhen18A for breeding hybrid varieties, pollination was carried out between Zhen18A plants and approximately 120 different rice germplasms. Approximately 85% of the F_1_ plants showed a superior per-plant yield compared to their parents, and some hybrids exceeded the yields of local control varieties by more than 10% (Chang et al. 2016). These results indicated that this technology is very promising for hybrid rice breeding, and thus it was named third-generation hybrid rice technology (TGHRT; Wang and Deng 2018).

### Further Research and Innovations in TGHRT

The developments of SPT in maize and TGHRT in rice broke the application bottleneck for GMS material, enabling the large-scale propagation of GMS lines for hybrid seed production. With these achievements as a foundation, researchers have conducted further research and development based on the technology, resulting in a series of remarkable achievements.

### Research on and Application of Recessive GMS Genes

The GMS genes suitable for construction of TGHRT are those that affect male fertility, but no other developmental aspects of the plant. Ideally, they are specifically expressed only in the male reproductive organ. Although many GMS genes have been cloned, some of them are expressed not only in the anther but also in other vegetative and/or reproductive tissues (Wan and Wu 2020; Abbas et al. 2021). The functions of these genes may extend beyond controlling pollen development, and mutations may have adverse effects on the growth and development of other tissues. Therefore, GMS mutants and their corresponding genes must be analysed in detail to ensure that the GMS genes are truly suitable for developing TGHRT.

Thus far, the following GMS genes have been successfully used for the construction of GMS maintainers: *OsMS26 (OsCYP704B2)*, *OsNP1*, *OsCYP703A3*, and *OsMS1 (PTC1)* in rice; *ZmMs45*, *ZmMs7*, *ZmMs26*, and *ZmMs30* in maize; *SlSTR1* in tomato; and *SiPKS2* in foxtail millet (Table 1). Some of these GMS genes, such as *OsMs26*, *OsNP1*, *ZmMs45*, and *ZmMs7*, were obtained through mutant gene cloning (Li et al. 2010, 2011; Yang et al. 2014; Chang et al. 2016; Wu et al. 2016). Others, including *OsCYP703A3*, *SlSTR1*, and *SiPKS2*, were acquired through gene editing (Du et al. 2020; Song et al. 2021; Zhang et al. 2023a). Many GMS mutants were obtained in model plants that are not necessarily suitable for hybrid breeding, although the corresponding GMS genes are ideal. To make use of these GMS genes, GMS lines can be generated in elite materials using CRISPR/Cas9-mediated gene knockout. The construction of the *OsCYP703A3*-based TGHRT is a good example of this strategy (Song et al. 2021). *OsCYP703A3* encodes a cytochrome P450 hydroxylase that is required for rice pollen wall formation. It is specifically expressed in anthers from the tetrad stage to binuclear microspore stage (Yang et al. 2014, 2018). The *OsCYP703A3* gene was originally identified in a GMS mutant *japonica* rice that was not suitable for hybrid breeding (Yang et al. 2014, 2018). To develop TGHRT in 9311, an *indica* rice that has many elite traits suitable for hybrid breeding, CRISPR/Cas9 technology was used to obtain the *OsCYP703A3* loss-of-function mutant *9311*^*03a3*^. Subsequently, a maintainer line (9311-3B) was constructed by transforming the *9311*^*03a3*^ mutant with the *OsCYP703A3* gene linked with the pollen-inactivation gene *orfH79* and the seed-sorting marker gene *DsRed2* (Song et al. 2021). Self-pollination of 9311-3B produced a male-sterile line (9311-3A) without the transgenic component and the maintainer line 9311-3B with the transgenic component in a 1:1 ratio. A hybrid rice developed from 9311-3A (Sanyou No.1) broke the yield record in China with a double-cropping yield of > 1,500 kg per mu (667 m^2^), representing a historic breakthrough in rice production (Song et al. 2021). These initial results demonstrate the immense application potential of TGHRT.

**Table 1:** Genes for developing the male maintainers and female maintainers in crops

Gene-ID	Gene	Encoding product	Biol. Function	Sterile type	Crop	References
Os03g0168600	*OsMS26 (OsCYP704B2)*	Cytochrome P450 protein	Lipid metabolism	Recessive, complete male sterility	*Oryza sativa*	(Li et al. 2010)
Os10g0524500	*OsNP1*	glucose-methanol-choline (GMC)oxidoreductase	Lipid metabolism	Recessive, complete male sterility	*Oryza sativa*	(Chang et al. 2016; Liu et al. 2017)
Os08g0131100	*OsCYP703A3*	Flavonoid 3-monooxygenase	Lipid metabolism	Recessive, complete male sterility	*Oryza sativa*	(Yang et al. 2014, 2018; Song et al. 2021)
Os09g0449000	*OsMS1 (PTC1)*	PHD-type transcription factor	transcription factor	Recessive, complete male sterility	*Oryza sativa*	(Li et al. 2011; Yang et al. 2019)
Zm00001d047859	*ZmMs45*	Strictosidin synthase	Lipid metabolism	Recessive,‘ complete male sterility	*Zea mays*	(Cigan et al. 2001; Chen et al. 2017)
Zm00001d020680	*ZmMs7*	PHD-finger transcription factor	Transcription factor	Recessive, complete male sterility	*Zea mays*	(Zhang et al. 2018a)
Zm00001d027837	*ZmMs26*	Cytochrome P450 monooxygenase	Lipid metabolism	Recessive, complete male sterility	*Zea mays*	(Djukanovic et al. 2013; Chen et al. 2017; Qi et al. 2020)
Zm00001d052403	*ZmMs30*	GDSL esterase/ipase protein	Lipid metabolism	Recessive, complete male sterility	*Zea mays*	(An et al. 2019)
Solyc03g053130	*SlSTR1*	putative strictosidine synthase	Lipid metabolism	Recessive, complete male sterility	*Solanum lycopersicum*	(Du et al. 2020)
Seita.2G112300	*SiPKS2*	plant-specific type III polyketide synthase	Lipid metabolism	Recessive complete male sterility	*Setaria italica*	(Zhang et al. 2023a)
Os05g0145000	*PTB1*	RING-type E3 ubiquitin ligase	Intrinsic E3 ligase activity	Female sterility	*Oryza sativa*	(Li et al. 2013; Xia et al. 2019)
Os02g0170300	*FST*	Bs-group MADS-box transcription factor	Transcription factor	Female sterility	*Oryza sativa*	(Yin and Xue 2012; Lee et al. 2013; Li et al. 2022)

### Research on and Innovations in Pollen-Inactivation Elements

Many countries have implemented a ‘zero tolerance’ regulatory policy towards transgenic crops, especially for food crops, such as rice and wheat. Therefore, the pollen-inactivation gene, which is tightly linked to the fertility-restoration genes, is a critical element to ensure tight containment of the transgene to the maintainer line. A perfect maintainer line for TGHRT should be capable of propagating male-sterile seeds without transmitting the transgenes (Cai et al. 2023). This requires the pollen-inactivation gene to be highly effective and lethal to the transgenic pollen, but not the non-transgenic pollen. Currently, the most widely used pollen-inactivation gene is the maize α-amylase gene *ZmAA1*, which causes the starch in pollen to be degraded, making the transgenic pollen unviable. However, the pollen lethality rate of α-amylase genes is somewhat affected by environmental factors. Studies have demonstrated that the transgene transmission rate through pollination ranges from 0.01 to 0.08% (Chang et al. 2016; Wu et al. 2016; Zhang et al. 2018). Therefore, it is necessary to enhance the efficiency of pollen-inactivation genes. There are two ways to achieve this goal. One is by using pollen-inactivation genes that are more effective than *ZmAA1*. The other is by increasing the expression levels of the available pollen-inactivation genes in transgenic pollen using stronger promoters. Combining pollen-inactivation genes may yield even better results.

Several genes besides *ZmAA1* have been reported to possess pollen-inactivation functions in various plants (Table 2). These include a new rice α-amylase (Huang et al. 2018), the cytotoxin barnase (Liu et al. 2014; Ma et al. 2015), the rice CMS-HL gene *orfH79* (Song et al. 2021), and the *Dam* gene (Zhang et al. 2018a). Among them, *orfH79* is a gametophytic CMS gene, which encodes a mitochondrial protein that induces pollen sterility in CMS-HL rice (Peng et al. 2010; Wang et al. 2013). ORFH79 fusion with the mitochondria-targeting signal peptide *RF1b* was expressed using the late-stage pollen-specific promoter *PG47*. The fusion protein was targeted to mitochondria, where it exhibited cytotoxic activity and disabled the transgenic pollen (Song et al. 2021). In this system, the transgene transmission rates through pollen were found to be 0.14–0.17% when the maintainer line was used to cross-pollinate the male-sterile line (Song et al. 2021). This value is more than double the transmission rate exhibited by the *ZmAA1* gene. Numerous other CMS genes have been identified from rice and other plant species (Wang et al. 2006; Itabashi et al. 2009; Luo et al. 2013; Toriyama 2021; Ren et al. 2022). Further research is warranted to determine whether these CMS genes can be used to improve pollen inactivation.

**Table 2:** Pollen inactivation genes and their pollen killing activities in the maintainers

Pollen inactivation gene	Mechanism	Transgenic pollen escape rate	Application crop	Reference
α-amylase gene *ZmAA1*	Degrade the starch in the pollen to inactivate pollen.	0.01 − 0.08%	Rice, Maize, foxtail millet	(Wu et al. 2016; Chang et al. 2016; Qi et al. 2020; Zhang et al. 2023a)
CMS-HL gene *orfH79*	The *orfH79* encoded protein is toxic to mitochondria and disables the pollen.	0.14 − 0.17%	Rice	(Song et al. 2021)
DNA adenine methyltransferase gene *Dam*	*Dam* encoded protein catalyzes the methylation of adenine residues in the pollen DNA, leading to pollen lethality.	Almost none	Maize	(Zhang et al. 2018a)
Unilateral cross-incompatibility (UCI) gene *ZmGa1F*	Proper pollen-specific *ZmGa1F* expression disrupts pollen tube growth and results in non-fertilization	Almost none	Maize	(Cai et al. 2023)

Zhang et al. (2018) introduced two pollen-inactivation genes, *ZmAA1* and *Dam* (encoding a DNA adenine methyltransferase), into the transgenic cassette of the multi-control sterility system (MCS) in maize to disrupt transgenic pollen. The *ZmAA1* gene was under the control of the *PG47* promoter, while the *Dam* gene was under the control of the pollen-specific promoter *Zm13*. Unger et al. (2001) showed that anther-targeted expression of the *Dam* gene can induce abnormal development of tapetal cells and microspores and render maize male-sterile. The transgenic pollen transmission rates of the MCS system co-expressing *ZmAA1* and *Dam* were 12% of the rate for the plants expressing *ZmAA1* alone in the same genetic background (Zhang et al. 2018a), indicating that co-expressing *ZmAA1* and *Dam* can significantly improve pollen inactivation. The effectiveness of this strategy is therefore worthy of testing in rice and other crops.

Recently, a novel SPT based on pollen tube inhibition (PTI) was developed in maize. It uses the maize unilateral cross-incompatibility (UCI) gene *ZmGa1F* as a ‘pollen killer’ to disrupt normal pollen tube development and prevent transgene transmission (Cai et al. 2023). UCI is a post-pollination reproductive barrier that unidirectionally prevents hybridisation when male and female parents are self-incompatible (Chen et al. 2022b; Zhang et al. 2023c). Gametophyte factor 1 (*Ga1*) is an intraspecific UCI system in maize and has been utilised in breeding (Zhang et al. 2018b). *ZmGa1F*, a pectin methylesterase gene located at the *Ga1* locus and expressed in pollen, confers the male function in the maize UCI system (Zhang et al. 2018b). Pollen-specific *ZmGa1F* expression did not affect pollen fertility, but disrupted pollen tube growth in the self-incompatible female, leading to no fertilisation (Cai et al. 2023). When *ZmGa1F* is expressed specifically in pollen, it severely inhibits pollen tube growth, which ultimately leads to no fertilisation. Examination of over 200,000 seeds revealed no seeds emitting fluorescence, indicating that the PTI-based transgene transmission rate is much lower than what has been reported for similar technologies. Thus, the method has great potential for successful application in commercial maize hybrid seed production (Cai et al. 2023). Meanwhile, it is worthwhile to investigate whether integration of *ZmAA1* and *Dam* with *ZmGa1F* can further decrease transgene transmission rates in rice.

Increasing the specific expression of pollen-inactivation genes in transgenic pollen with stronger promoters is an alternative approach to preventing transgene transmission. In previously reported systems, pollen-inactivation genes, including the maize α-amylase gene *ZmAA1*, the maize unilateral cross-incompatibility gene *ZmGa1F*, and the rice CMS gene *orfH79*, were all expressed using the maize pollen-specific promoter *PG47*. The *PG47* promoter is derived from a maize polygalacturonase gene that is specifically expressed in mature pollen grains from the first mitosis (stage 11) until pollen maturity (stage 12) (Allen and Lonsdale 1993). Rice and maize pollen grains begin to accumulate starch at stage 11 and form numerous large starch granules by maturity (Chang et al. 2016; Zhang et al. 2018a). The *PG47* promoter driving the *ZmAA1* gene can effectively prevent the formation of starch granules in pollen grains carrying the transgene, rendering the pollen inactive in both maize and rice transgenic plants (Wu et al. 2016; Chang et al. 2016; Zhang et al. 2018a).

Despite the satisfactory results obtained with the *PG47* promoter, to avoid potential intellectual property issues during commercial production, Wang et al. (2020) identified more promoters that were specifically and strongly expressed in late-stage rice pollen grains. They conducted RNA sequencing analysis to identify genes that were specifically expressed in mature pollen grains, followed by quantitative reverse transcription-polymerase chain reaction analysis to determine the relative RNA expression levels of these genes in anthers at various developmental stages. Six *OsLSP* gene promoters were identified that showed high levels of RNA expression. The top three promoters (*OsLSP3*, *OsLSP5*, and *OsLSP6*) were found to be highly active at stage 11 and able to effectively drive *ZmAA1* to inactivate pollen in rice. However, the *OsLSP4* promoter, which had higher activity at stage 12, but lower activity at stage 11, did not drive *ZmAA1* to inactivate pollen, indicating that strong promoter activity during stage 11 is crucial for constructing the pollen-inactivation system. The *OsLSP3*, *OsLSP5*, and *OsLSP6* promoters provide additional tools for TGHRT (Wang et al. 2020). Combining different pollen-inactivation genes with these promoters may improve pollen inactivation.

## Research on and Innovation in Seed-Sorting Elements

Efficient, accurate, and low-cost sorting of maintainer and GMS lines is an essential part of TGHRT. Most maintainer lines use the red fluorescence protein genes *DsRed2* and *mCherry* as marker genes for seed sorting (Table 3). Under 565-nm light excitation conditions, the maintainer seeds with these reporter genes produce red fluorescence in the aleurone layer, so they can be distinguished from the non-fluorescent GMS seeds that do not contain the transgene. However, seeds produced in the field are easily influenced by environmental and cultivation conditions, such as pests and diseases, fluctuations in temperature and humidity, and water and fertiliser management. As a result, some seeds may develop poorly, or the seed coats may develop brown or black spots. The poorly developed maintainer seeds usually produce weak fluorescence, and brown or black spots on the seed coat can produce a weak fluorescence signal, even though the seeds do not carry the transgene. Both of these effects can influence the accuracy of seed sorting, which ultimately reduces the purity of the GMS seeds. In practical applications, if the purity of GMS seeds cannot be guaranteed, subsequent hybrid seed production and farm planting will face the risk of transgenic contamination. Furthermore, seed sorting based on red fluorescence requires an efficient and precise sorting machine, but currently available fluorescence-sorting machines can only process 35 kg seeds per hour (Song et al. 2021). To reduce reliance on the fluorescence sorter, cross-pollination of the GMS line with the maintainer line is used to propagate the GMS seed, but this increases the labour required for seed production. Therefore, innovation of seed-sorting elements in TGHRT is important.

**Table 3:** Seed sorting genes for GMS maintainer

Gene/ protein	Mechanism	Sorting method	Detection equipment	Application crop	Reference
Fluorescent protein *DsRed2*/ mcherry	Under 565 nm excitation light conditions, the fluorescent protein emits red fluorescence.	Color-based seed sorting method	Fluorescent sorting machine	Rice, Maize	(Wu et al. 2016; Chang et al. 2016; Qi et al. 2020)
AGP synthesis protein	Using *amiRNAs* to suppress AGP gene expression interfered with endosperm starch synthesis.	Weight-based seed sorting method	Weight-sorting machines	Rice	(Wu et al. 2021)
Anthocyanins synthesis gene *ANT1*	Induce transgenic seedlings to accumulate a greater quantity of anthocyanins, thereby making them purple.	Color-based seedling sorting method	Naked eye	Tomato	(Du et al. 2020)
Herbicide-resistant *Bar* gene	*Bar* gene makes plants no longer sensitive to glufosinate by changing the metabolic pathway of plants.	Herbicide-resistant based seedling sorting	Sprayer	Maize	(An et al. 2020)
Betalain biosynthesis gene *RUBY*	Betalains are a type of natural plant pigment that is bright red and easily visible to the naked eye.	Color-based seed sorting method	Naked eye	Foxtail millet	(Zhang et al. 2023a)

Wu et al. (2021) effectively suppressed the expression of genes encoding ADP-glucose pyrophosphorylase, which are essential for the biosynthesis of endosperm starch, by expressing artificial microRNAs (amiRNAs) in the endosperm. The grain weight of seeds carrying the transgene was significantly lower than that of the wild-type seeds, but seed germination and growth in the field were unaffected. The amiRNA expression cassette was then linked with the male fertility gene *OsNP1* and the pollen-inactivation gene *ZmAA1* and transformed into the *osnp1* mutant to construct a weight-based seed-sorting system for the TGHRT (Table 3). In this system, the GMS seeds possess normal endosperm and are heavier, whereas the maintainer seeds have shrunken endosperms and are lighter. The GMS seeds can be conveniently and precisely separated from the maintainer seeds using a weight-sorting machine, thus ensuring the availability of pure and fully filled GMS seeds (Wu et al. 2021). The weight-based sorting machines can process 3,000–6,000 kg of rice seeds per hour (Wu et al. 2021), a sorting efficiency approximately 100 times higher than the fluorescence sorter. Thus, self-pollination of maintainer lines can be used for propagation of the GMS lines. This method is suitable for mechanised production and can significantly reduce the labour and costs of producing GMS seeds.

Recently, Zhang et al. (2023a) established an SPT system in foxtail millet using the betalain biosynthesis gene *RUBY* as the selection marker (Table 3). Betalain is a type of natural plant pigment synthesised from tyrosine (He et al. 2020). The pigment is bright red and easily visible to the naked eye. The two types of seeds can therefore be distinguished by the naked eye with no need for any equipment or dehulling. The SPT maintainer line can also be identified in the field based on the pigmentation of the plant. Plant pigmentation is a useful trait for identifying the maintainer line in the field and can aid the cleaning of contaminated maintainer lines from the GMS lines during hybrid seed production. It is a valuable sorting marker for crops that do not have an efficient pollen-inactivation system and cannot rely on cross-pollination by the maintainer line for propagation of a pure GMS line. This strategy has been tested in tomato by Du et al. (2020), who applied the tomato anthocyanin synthesis gene *ANT1* as a sorting marker. They connected the *ANT1* gene with the male fertility gene *SlSTR1* and introduced it into the corresponding male-sterile mutant. *ANT1* induced the transgenic seedlings to accumulate more anthocyanins (Mathews et al. 2003), thereby enabling a clear differentiation between the male-sterile and maintainer lines based on seedling colour. However, sorting by leaf colour is not efficient for the production of hybrid rice and other cereal crops.

In addition to seed colour and weight-based sorting, An et al. (2020) devised a system that can be used to select the maintainer line using the herbicide-resistant *Bar* gene (Table 3). They constructed a T-DNA cassette that included a fertility-restorer gene, pollen-inactivation gene, colour-selection gene, and the *Bar* gene, and transformed it into the maize male-sterile mutant *ms7* to generate a maintainer line. The GMS line produced by maintainer self-pollination was selectively killed by herbicide treatment, leaving the pure maintainer line. The GMS line was propagated by cross-pollinating with pollen from the maintainer line.

## Construction of Male-Sterile Line and Maintainer Line in One Step

Typically, the construction of a TGHRT system involves two steps. The first is to obtain a GMS mutant through either mutagenesis or genome editing. The second is to transform the GMS mutant with the TGHRT transgene cassette. However, with an efficient genetic transformation system, a GMS maintainer line can be quickly constructed by CRISPR/Cas9 knockout of the wild-type GMS gene and introduction of the maintainer transgene through a single transformation.

Qi et al. (2020) tested this strategy in maize by constructing two plant transformation vectors. One vector contained the CRISPR/Cas9 gene editing tool targeting two introns of the maize male-sterility gene *ZmMS26*. This vector was able to delete an exon of *ZmMS26* to create the GMS mutation. The second vector contained *ZmMS26* cDNA under the *ZmMS26* native promoter, the *ZmAA1* gene under the *PG47* promoter, and the *DsRed* gene under the *LTP2* promoter. A plant co-transformed with the two vectors was found to have homozygous mutation of the *ZmMS26* gene and the maintainer cassette. The plant was fertile and set non-fluorescent seeds and fluorescent seeds. The fluorescent seeds grew into fertile plants, while the non-fluorescent seeds grew into male-sterile plants. The fertile plants carrying the homozygous *zmms26* mutation, but lacking the CRISPR/Cas9 cassette, were identified by PCR and saved as the maintainer line.

This strategy was also tested by Zhang et al. (2023a) in foxtail millet. They constructed two vectors. A CRISPR/Cas9 vector was designed to knock out *SiPKS2*, a homologue of *OsPKS2* that is involved in the formation of the pollen wall. The second vector contained functional *SiPKS2* linked with the pollen-inactivation gene *ZmAA1* and the seed-colour gene *RUBY*. The two vectors contained different transformation selection markers; one contained the *HPT* gene, and the other contained the *NPT II* gene. Synonymous codons in exogenous *SiPKS2* were replaced to avoid CRISPR/Cas9-mediated knockout of the transgene. GMS lines and GMS maintainer lines were simultaneously generated. This method is convenient for the *de novo* construction of new GMS maintainer lines and can be applied to rice.

### Development of Female-Sterile Lines for the TGHRT

Currently, transplanting is the method most commonly used to produce hybrid rice seeds. In this system, parents are transplanted in alternate rows (usually five or six rows of the male-sterile line and one row of the paternal line) to facilitate cross-pollination. Human assistance is required to shake the pollen grains into the air to increase cross-pollination. Paternal lines are then manually removed to prevent paternal seeds mixing with hybrid seeds during harvest (Tang et al. 2020). This method is labour-intensive, and it is also impossible to ensure complete removal of the paternal plants. The labour-intensity of the production of hybrid rice seeds has long been the major problem hindering the application of hybrid rice (Ma and Yuan 2015; Xia et al. 2019). Reducing the labour requirement for hybrid seed production is an effective way to improve the market competitiveness of hybrid rice. If the paternal line is female-sterile, then the seeds of the two parental lines can be mixed for direct seeding using a machine, without the need to remove the paternal line after pollination. This would greatly reduce the labour cost and increase the efficiency of hybrid seed production.

A TGHRT strategy can be used to propagate female-sterile lines if the GMS mutant and the GMS gene are replaced by a female-sterile mutant and the corresponding female-fertility gene. This strategy was first tested by Xia et al. (2019), who constructed a transgene cassette containing the female fertility-restoration gene *POLLEN TUBE BLOCKED 1* (*PTB1*), the pollen-inactivation gene *ZmAA1*, and the red fluorescence protein gene *DsRed2*, and introduced it into the wild-type paternal line MH86. *PTB1* is a key maternal sporophytic gene encoding a RING-type E3 ubiquitin ligase, which is essential for pollen tube growth and female fertility in rice (Table 1) (Li et al. 2013). The transgenic plants were crossed with a *ptb1* mutant and then self-pollinated to obtain progeny carrying the hemizygous *PTB1* transgene and homozygous *ptb1* mutation (the female-sterility maintainer). During self-pollination, pollen grains carrying the transgene were inactivated, and pollen grains not carrying the transgene fertilised the female gametes, leading to the production of 50% *ptb1* mutant seeds and 50% transgenic seeds that were sorted based on red fluorescence. Using this approach, a female maintainer line capable of self-reproduction was created, and propagation of the female sterile line was achieved (Xia et al. 2019). However, the functioning of *PTB1* is affected by the ambient temperature. The *ptb1* mutant is not completely sterile and has a seed-setting rate of 1.8% (Li et al. 2013). Thus, this system cannot be employed for commercial production. A mutant with complete female sterility could solve this problem.

The MADS-box gene FEMALE-STERILE *(FST)* is genetically sporophytic and plays a key role in regulating chalaza formation, integument morphogenesis, and the early development of the zygotic embryo and endosperm (Table 1) (Yin and Xue 2012; Lee et al. 2013; Li et al. 2022). The *fst* mutation confers complete female sterility in various genetic backgrounds under different environmental conditions without affecting vegetative growth or pollen viability. Therefore, the *fst* mutant has great potential for commercial application. Using the female sterility gene *FST* and its mutant *fst*, Li et al. (2022) attempted to establish a female maintainer line for propagating female-sterile seeds. They constructed a linkage expression vector consisting of *FST* genomic DNA and the *BTZ*-RNAi expression cassette. *BTZ*-RNAi inhibits the expression of the *CYP81A6* gene, which can make transgenic rice sensitive to the herbicide bentazon (*BTZ*^S^). This *BTZ*^S^ trait can be used as a marker to eliminate the transgenic seedlings through herbicide treatment (Pan et al. 2006; Wang et al. 2012; Lu et al. 2017). The construct was transformed into the homozygous *fst* mutant to obtain hemizygous *FST*^*T*^ T_0_ plants. The presence of T-DNA in the transgenic plants restored female fertility. Self-pollination of the transgenic plants produced 3:1 segregation of progeny with and without the transgene. Seedlings with the transgene were killed by bentazon application, leaving a pure, transgene-free female-sterile line (Li et al. 2022). However, this system has a problem in continuing propagation of the maintainer line, because each generation after self-pollination, the ratio of maintainer seeds is reduced in the population, which will eventually exhaust the maintainer line.

## Future Challenges and Opportunities for TGHRT

The first two generations of hybrid rice technologies have contributed significantly to the global food supply since their deployment. However, after reaching a peak in the 1990s, the cultivation area of hybrid rice gradually declined from 65% to 45% of the total rice acreage by 2019 (Wang 2020; Huang 2022). The main reasons for the reduction were stagnant yield improvement, the extensive labour requirement for hybrid seed production, and the poor eating quality of many hybrid varieties, which make hybrid rice cultivation unprofitable. These problems are all rooted in the limitations of traditional male-sterile systems, which impede progress in breeding superior hybrid varieties. Overcoming these problems requires technical breakthroughs that can overcome the limitations of traditional technologies and accelerate the improvement of parental lines.

The establishment of TGHRT is a significant breakthrough in the field of hybrid rice breeding. Compared with traditional technologies, TGHRT has the following advantages. First, as the GMS line is controlled by a recessive nuclear gene, any rice germplasm harbouring the wild-type gene can restore the fertility of the F_1_ hybrid. Consequently, TGHRT enables a wider range of germplasms to be selected as paternal lines for breeding hybrids with superior heterosis. Second, the sterility of the GMS line is stable and insensitive to environmental conditions, which eliminates the risks associated with unpredictable climate changes and enables safe hybrid seed production regardless of weather conditions. Third, as TGHRT enables recessive female sterile lines to be propagated, a mechanised mixed-seeding and mixed-harvesting approach can be used for hybrid seed production, which significantly reduces the labour requirement and increases efficiency. Fourth, the sterility and fertility-restoration traits of TGHRT are each controlled by a single genetic locus, which allows stable inheritance in different genetic backgrounds. Both loci can be easily transferred through marker-assisted breeding, making it easier to breed new maintainer lines. This will accelerate improvements of male and female parental lines. Fifth, because the fertility-restoration gene is linked to the pollen-inactivation gene, it blocks the transmission of transgenic components through pollen and enhances the environmental safety of TGHRT. Finally, although TGHRT is based on transgenic technology, only the maintainer line contains the transgene. The commercial male and female parental lines, as well as the hybrid seeds, are all transgene-free, meaning that commercial products of TGHRT do not need transgenic surveillance.

While TGHRT has significant technical advantages over traditional CMS and EGMS systems, full exploitation of this technology in agricultural production still faces significant challenges in the development of elite parental lines that can be used to breed superior hybrid varieties. Thus far, satisfying results have only been reported for the *OsNP1*-based Zhen18B and *OsCYP703A3*-based 9311-3B systems (Song et al. 2021). This is far from meeting the need for hybrid rice varieties that can be grown in broad geographical areas and diverse environmental conditions. The two reported female-sterility maintainer systems both have technological defects that make them unsuitable for commercial breeding (Xia et al. 2019; Li et al. 2022). Further technical innovations are required to overcome these defects.

Approximately 49 GMS genes and 6 female-sterility genes have been identified in rice (Wan and Wu 2020; Shen et al. 2021). Some of these genes are specifically expressed in the male or female reproductive organs, and mutants of these genes are stable under different environmental conditions and do not have a negative impact on the development of other tissues. These genes are excellent candidates for TGHRT. In addition to these rice genes, many recessive male and female sterility genes have been identified in other plant species, such as maize and *Arabidopsis* (Gómez et al. 2015; Wang et al. 2022). Because genes regulating male and female fertility are often functionally conserved across different plant species, the homologues of these genes can also be tested in rice. Decades of traditional rice breeding have led to the accumulation of numerous rice germplasms with excellent agronomic traits. With many fertility-controlling genes cloned, *de novo* construction of new maintainer systems can be performed with the available rice germplasms.

Several factors should be considered when selecting rice germplasms for *de novo* construction. First, it is necessary to use elite rice lines with high stigma exsertion and high seed-setting rates for construction of the male-sterile system, and elite rice lines with large anthers and a high pollen load for construction of the female-sterile system, because these reproductive traits are necessary for a high yield of hybrid seeds (Li et al. 2007; Cheng et al. 2007; Huang et al. 2014; Zhu 2016). In addition, strong combining ability is required because this will allow more superior hybrid varieties to be generated by pairing with other germplasms. These traits are not only valuable for the *de novo* maintainer, but they will also accelerate the breeding process when the *de novo* maintainer is used for the cross-breeding of new maintainer lines.

Although *de novo* construction is a quick way of developing a new maintainer, cross-breeding is probably a better choice if an established maintainer is available. This is especially true if one considers the issues of transgenic management. According to China’s current policy for transgenic management, a new *de novo* maintainer must go through three stages of testing (an intermediate test, an environmental release test, and a production test) before a safety certificate will be granted (Li et al. 2014; Liang et al. 2022). The testing process requires extensive experiments, a large budget, and a minimum of 4 years before safety certificate application. Once the safety certificate is obtained, new varieties with the same transgenic event do not need to go through the testing process. Cross-breeding usually takes approximately six generations (~ 3 years) to obtain a stable line, and the cost is low. In this regard, development of new maintainer lines with a certified transgenic event through cross-breeding is certainly a better choice than *do novo* construction. Currently, the *OsNP1*-based Zhen18B has passed 2 years of production testing in China and is on the way to safety certificate application (Liao et al. 2021). By crossing Zhen18B with commercial EGMS lines, CMS maintainers, and elite inbred lines with outstanding out-crossing traits, a number of maintainer lines with a higher yield of hybrid seeds, excellent combining abilities, good grain quality, and enhanced resistance to rice blast and rice blight diseases have been bred that have passed China’s national standard for commercialisation (Liao et al. 2021). Several hybrids derived from these *OsNP1*-based maintainers are in the national variety approval test in China (Liao et al. 2021). Nonetheless, breeding of more maintainer lines with further improvement of various agronomic traits is still urgently needed.

Decades of molecular genetics and functional genomics research have led to the accumulation of abundant knowledge of genes controlling various desirable agronomic traits in rice, such as high grain yield, high grain quality, aroma, wide adaptability, resource efficiency, early maturity, stress tolerance, resistance to diseases and pests, tolerance to barrenness and thinness, and lodging resistance (Chen et al. 2022a). Genes regulating stigma traits important for hybrid rice breeding have also been reported. Using a genome association assay technique, Dang et al. (2020) identified a novel gene, *OsSYL2*, for increasing style length in rice. Zhu et al. (2023) found that simultaneous knockout of *GS3*, *GW8*, and *GS9* could effectively increase stigma exsertion and the out-crossing rate without negative impacts on other agronomic traits. These loci can be explored for breeding GMS lines with higher out-crossing rates and higher yields of hybrid seeds. It is known that *indica/japonica* hybrids have stronger heterosis than *indica/indica* and *japonica/japonica* hybrids. However, hybrid sterility of the inter-subspecies F_1_ poses a barrier to their application (Qian et al. 2016; Liu et al. 2020). Zhou et al. (2023) constructed and assembled different combinations of naturally compatible alleles of four loci, *S5*, *Sc*, *pf12*, and *f5*, and found that pollen and embryo sac fertility was fully recovered in some F_1_ test-crosses. This result revealed a breeding scheme that may facilitate the breeding of inter-subspecies hybrid varieties (Zhou et al. 2023). It is crucial that rice breeders fully utilise the accumulated knowledge and apply modern technologies, such as genome sequencing, molecular marker-assisted selection, and gene editing to accurately identify, create, and assemble these desirable traits into parental lines for the breeding of superior hybrid varieties. Fully exercising these technical advances will accelerate the application of TGHRT to meet the current demand for hybrid rice production and contribute to the early achievement of goals related to high-yield, high-quality, high-efficiency, and environmentally friendly agricultural production (Hernández-Soto et al. 2021; Yu et al. 2022).

In summary, the TGHRT system has multiple advantages over the traditional three-line and two-line systems for hybrid rice breeding and production. It is an excellent example of how advances in biotechnology can help address some of the challenges of crop breeding. With continuous research and development, TGHRT will play an increasingly important role in the utilisation of heterosis in rice. Similar technologies could be developed for other crops, especially wheat, sorghum, soybean, rapeseed, and many others. Almost 200 male-sterile genes have been cloned and identified in *Arabidopsis*, rice, maize, soybean, wheat, and other plants (Wan et al. 2021). With the availability of CRISPR/Cas9 technology and the cloning of numerous male-sterile genes, it is becoming increasingly convenient to establish technologies analogous to TGHRT in these crops. Because male-sterility genes are mostly conserved across different plant species, this strategy could also be applied to other crops, particularly those in which heterosis has not been exploited due to a lack of CMS and EGMS lines. GMS was first applied to commercial hybrid maize production in the United States in 2012, and the GMS lines and hybrid varieties generated by this technology have been successively deregulated by the United States, Japan, and Australia (Wu et al. 2016; Liao et al. 2021). Rice is the most important staple crop in China. Currently, the GMS lines and hybrid combinations derived from TGHRT are still regarded as transgenic products in China. Nonetheless, China has gradually adjusted its gene editing technology and transgenic technology for crops such as soybeans and corn (Liang et al. 2022). It is expected that TGHRT, once authorised for commercial application, will contribute significantly to rice production and food security in China and globally.

## Data Availability

Not applicable.

## Abbreviations

CMS: cytoplasmic male sterility
EGMS: environment-sensitive genic male sterility
TGHRT: Third-generation hybrid rice technology
GMS: genic male sterility
CMS-WA: CMS-Wild abortive
CMS-HL: CMS-Honglian SPT:Seed Production Technology
HHZ: Huanghuazhan
PTI: pollen-tube-inhibition
UCI: unilateral cross-incompatibility
AGP: ADP-glucose pyrophosphorylase
